# Tumor Theranostics of Transition Metal Ions Loaded Polyaminopyrrole Nanoparticles: Erratum

**DOI:** 10.7150/ntno.67620

**Published:** 2022-02-18

**Authors:** Shuyao Li, Shuwei Liu, Lu Wang, Min Lin, Rui Ge, Xing Li, Xue Zhang, Yi Liu, Lening Zhang, Hongchen Sun, Hao Zhang, Bai Yang

**Affiliations:** 1 State Key Supramolecular Structure and Materials Laboratory, College of Chemistry, Jilin University, Changchun 130012, P. R. China;; 2 The Oral Pathology Department, School and Hospital attached to Stomatology, Jilin University, Changchun 130021, P. R. China;; 3 Collaborative Innovation Center attached to Marine Biomass Fibers, Shandong Province Materials and Textiles, Marine Biobased Materials Institute, Materials Science and Engineering School, Qingdao University, Qingdao 266071, P. R. China;; 4 Department of Thoracic Surgery, China-Japan Union Hospital, Jilin University, Changchun 130033, P. R. China.

In the original paper 1 on p. 218, Figure 6E there is error in the fluorescence image of PI and FDA co-staining cells, which arises from confusion of the images. In the revised Figure 6 shown below, the correct fluorescence images are provided. In addition, on p. 219, Figure 8D there is error in the tumors photograph of laser only group, which arises from confusion of the images. In the revised Figure 8 shown below, corrected images are provided by repeating the entire animal experiment. To ensure that the animal experiments are contrasted using the same batch of mice with Figure 8, the corresponding characterizations in Figure S9, S10 and S14 are also included with this correction. These corrections do not alter the major conclusions of this article.

In addition, part of the description was incorrect. In "Animal experiments" (p. 213 right lines 2-10), the correct description should be “4 weeks' old balb/c nude mice (weighing ~ 18 g) were bought from Beijing Vital River Laboratory Animal Technology Co. Ltd. The mice were used under protocols approved by Jilin University Laboratory Animal Center. After one week's feed, 2×10^6^ of KB cells dispersed in 150 μL of cell culture were injected subcutaneously into the right back leg of the mice”. In "Results and Discussion" (p. 216 right line 14), the correct description should be “As exposed under laser irradiation for 0, 3, 8, 10 min”. These corrections do not alter the major conclusions of this article. Besides, for the laboratory animal welfare principle of “3R (Reduction, Replacement, Refinement)”, we shared a blank control group with another work of our group in MRI (Figure 7G-J), which is quoted here 2.

## Figures and Tables

**Figure 6:**
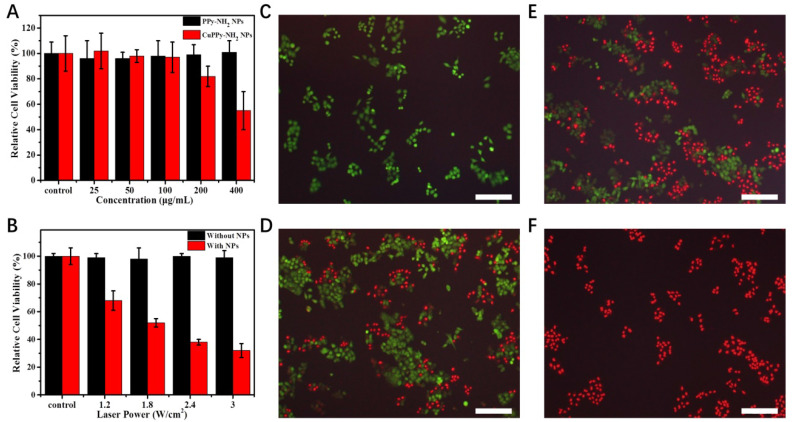
(A) The toxicity of KB cells with CuPPy-NH_2_ NPs and PPy-NH_2_ NPs in different concentration. (B) KB cells are incubated with or without 50 μg/mL CuPPy-NH_2_ NPs for 30 min, and then they are irradiated by an 808 nm laser with the power density of 1.2, 1.8, 2.4 and 3 W/cm^2^ for 8 min. Fluorescent images of PI and FDA co-staining cells after combined therapy for 0 (C), 3 (D), 8 (E) and 10 min (F), respectively. The scale bar in (C-F) represents 200 μm.

**Figure 8:**
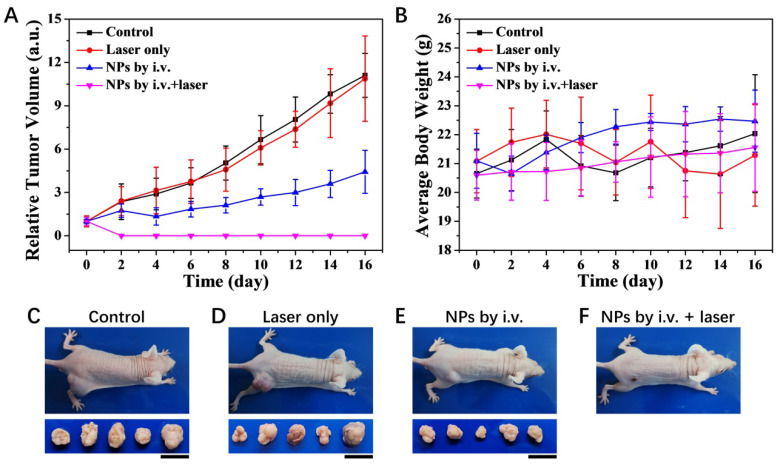
Photothermal therapy of KB tumors *in vivo*. (A) Relative tumor volume growing trend. (B) Average body weight for each group. (C-F) Photographs of typical mouse bearing tumor model and tumors taken from each group in the 16th day. The mean tumor volume in each group was 89.4, 90.7, 91.4 and 92.3 mm^3^ at the beginning of the treatment and 992.7, 986.5, 404.7 and 0 mm^3^ at the end of the treatment, respectively. The scale bar for tumors represents 20 mm.

**Figure A1:**
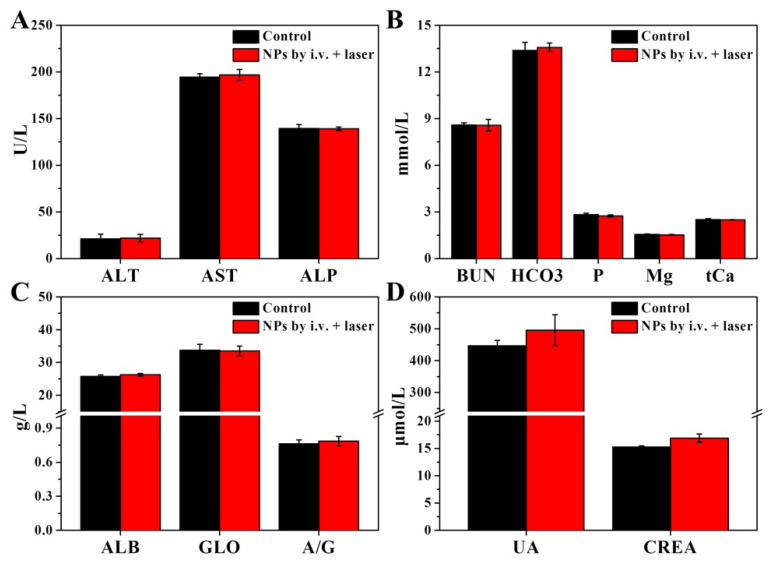
** S9.** Liver and renal functions tested 24 h post i.v. injection of CuPPy-NH_2_ NPs. All of the parameters are in normal scale comparing to the healthy control. (A) Alanine aminotransferase (ALT), aspartate transaminase (AST) and alkaline phosphatase (ALP). (B) Blood urea nitrogen (BUN), dicarbonate (HCO_3_), serum phosphorus (P), serum magnesium (Mg) and total calcium (tCa). (C) Albumin (ALB), globulin (GLO) and the ratio of ALB to GLO (A/G). (D) Uric acid (UA) and creatinine (CREA).

**Figure A2:**
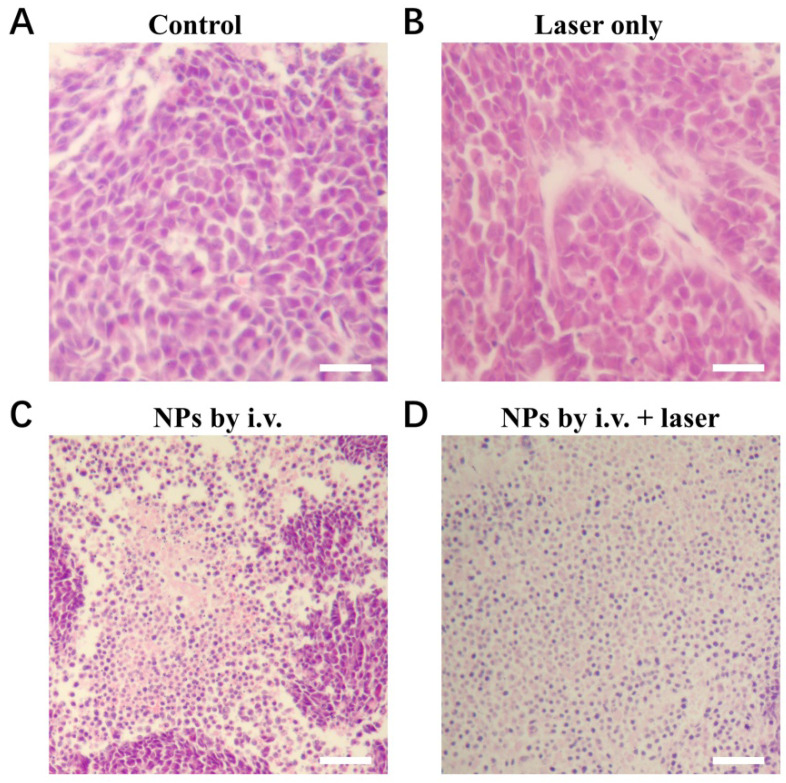
** S10.** H&E stained tumor slices after 16 days of treatment. (A) Control group. (B) Laser only group. (C) CuPPy-NH_2_ NPs only group. The tumors were removed at 16 days in all three groups. (D) CuPPy-NH_2_ NPs + laser group. In (D), after photothermal treatment with NIR laser, the tumor was removed immediately. The scale bar is 100 µm.

**Figure A3:**
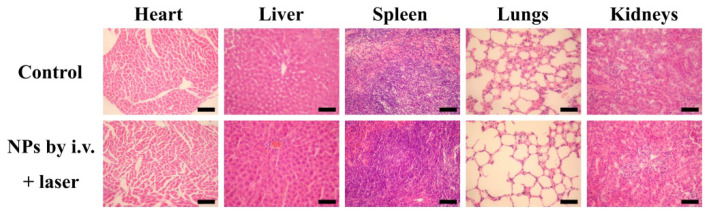
** S14.** H&E stained splanchnic slices after 16 days of treatment. The control group is age-matched healthy mice. The NPs by i.v. + laser group is thermo-chemotherapy group in our experiments. The scale bar is 100 µm.
